# Isolation, characterization and whole-genome analysis of a potentially novel strain of duck hepatitis A virus type 3 from a vaccinated duck flock in China

**DOI:** 10.3389/fmicb.2026.1775404

**Published:** 2026-03-25

**Authors:** Yuqing Dan, Linlin Li, Haoqian Wang, Shuhui Sang, Bingqian Zhou, Zeng Wang, Xia Yang, Xinwei Wang

**Affiliations:** College of Veterinary Medicine, Henan Agricultural University, Zhengzhou, China

**Keywords:** duck hepatitis A virus type 3, genomic analysis, isolation and identification, VP1, whole-genome analysis

## Abstract

**Introduction:**

Duck viral hepatitis (DVH) is an acute infectious disease caused by duck hepatitis virus. It is characterized by hepatic hemorrhage, hepatomegaly, and opisthotonos in ducklings, posing a significant threat to the global poultry industry.

**Methods:**

In this study, a potentially novel duck hepatitis A virus type 3 (DHAV-3) strain, designated HNAY2024, was isolated and purified from an immunized duck farm in Henan Province. The complete genome sequence was obtained through whole-genome sequencing and subjected to phylogenetic analysis. Meanwhile, in-depth analysis of the VP1 gene was performed to determine its genotype, and key mutation sites were identified through amino acid sequence alignment.

**Results:**

The HNAY2024 strain demonstrates vaccine-escape potential and breaks the classical pattern where DHAV-3 primarily causes high mortality in ducklings under one week of age. Whole-genome sequencing and phylogenetic analysis revealed that HNAY2024 shares 91.9%–99.9% nucleotide identity with other DHAV-3 reference strains and clusters most closely with the Egyptian strain ZU-ARMY-DHA-36, the Henan strain HNXY23, and the Shandong strain WKX03, indicating its potential dissemination to possibly broader regions. Further analysis of the VP1 gene confirmed that HNAY2024 belongs to genotype I (GI) of DHAV-3. Amino acid alignment identified eight substitutions (V413M, E683Q, V855I, F892S, S1149I, T1151S, E1519G, and K1956E).

**Conclusion:**

The eight identified amino acid mutations are predicted to contribute to adaptive changes in viral antigenicity, replication fitness, and host interactions. Such alterations could influence the viral host range or transmission dynamics, thereby posing potential ecological risks for both domestic poultry and wild birds.

## 1 Introduction

Duck Viral Hepatitis (DVH) is an infectious disease of significant clinical and economic concern in the global duck industry, characterized pathologically by acute hepatitis and high mortality in young ducks (Ren et al., 2012; Niu et al., 2019). Historically, the term “DVH” has been used broadly to describe a hepatitis syndrome caused by multiple hepatotropic viruses. Duck hepatitis A (caused by DHAV) is one of the hepatitis syndromes (Ou et al., 2022).

Duck hepatitis A virus (DHAV), a member of the genus *Avihepatovirus* within the family *Picornaviridae*, is the most prevalent etiological agent. Based on genetic and antigenic divergence, DHAV is further classified into three major genotypes (Kim et al., 2007a; Ou et al., 2022): DHAV-1, the classical and globally distributed virulent genotype; DHAV-2, which appears largely confined to Taiwan, China; and DHAV-3, a genotype predominantly endemic to East Asia, particularly in eastern China and South Korea (Annamalai et al., 2022; Kim et al., 2007a; Wang et al., 2008; Lefkowitz et al., 2018; Zhou et al., 2022). DHAV-3 exhibits distinct regional endemicity, frequently co-circulates with DHAV-1 leading to co-infections, and demonstrates high pathogenicity in ducklings (Niu et al., 2019; Yehia et al., 2021; Ou et al., 2022). These attributes make DHAV-3 a key genotype targeted for surveillance and control in Asian waterfowl production.

Based on the VP1 gene, DHAV-3 strains segregate into two primary genotypes and an independent group: Genotype I (GI), also designated the CH group, which contains nearly all Chinese isolates; and Genotype II (GII), referred to as the KV group, consisting of Vietnamese wild strains and Korean isolates; and a distinct E group, predominantly consisting of Egyptian isolates. Furthermore, Genotype II (KV group) can be subdivided into two distinct lineages: Subtype S1 (K group), primarily composed of Vietnamese wild strains, and Subtype S2 (V group), which encompasses the Korean strains (Xu et al., 2013; Zhang et al., 2017; Hassan et al., 2020; Yehia et al., 2021).

In mainland China, DHAV-1 and DHAV-3 are the predominant genotypes (Liu et al., 2019; Zhao et al., 2024). However, since 2013, DHAV-3 has become predominant in Chinese duck populations (Liu et al., 2021), and it has now become one of the most important pathogens affecting the healthy development of the duck industry in China (Cao et al., 2020).

The DHAV genome is a single-stranded RNA molecule of approximately 7.8 kb, featuring a 5′ untranslated region (5′UTR), a single large open reading frame (ORF), and a 3′UTR (Li et al., 2013; Rohaim et al., 2021). The ORF encodes a polyprotein that is co- and post-translationally processed by viral proteases (protease 2A and 3C). Initial cleavage by protease 2A yields three precursors: P1 (structural proteins), P2, and P3 (non-structural proteins) (Kim et al., 2007a; Kim et al., 2006; Kristensen and Belsham, 2019). Subsequent proteolytic processing by protease 3C generates the mature proteins. The P1 precursor is cleaved to yield the structural proteins VP0, VP3, and VP1. Processing of the P2 and P3 precursors produces the non-structural proteins 2A1, 2A2, 2A3, 2B, 2C, and 3A, 3B, 3C, 3D, respectively (Probst et al., 1998). The P1 precursor is cleaved to yield the viral capsid, which harbors the major antigenic epitopes and mediates receptor recognition. The P2 precursor is involved in the primary cleavage of the polyprotein and facilitates viral RNA synthesis. The P3 precursor is processed into mature proteins and orchestrates viral RNA replication (Kolupaeva et al., 1996; Kim et al., 2007b). The VP1 protein of DHAV harbors critical antigenic determinants and is closely associated with viral antigenicity, pathogenicity, and evolution, making it the molecular basis for serological and genetic classification (Rohaim et al., 2021; Ma et al., 2015; Liu et al., 2008; Hisham et al., 2020; Wen et al., 2015).

This article reported that a prevalent DVH virus in one field originated from a farm in Henan Province. Ducks on this farm were suspected of being infected with DVH after DVH vaccination or passive immunization using duck hepatitis antibodies, and its genome was comprehensively analyzed and evaluated. This study elucidates the molecular epidemiology and antigenic evolution of this potentially novel vaccine-escape DHAV-3 variant, thereby providing essential reference materials for updating vaccine immunization strategies and diagnostic methods.

## 2 Materials and methods

### 2.1 Materials

Fifteen-day-old non-SPF goose embryos were sourced from Yuanyang Guoxiu Farm, Henan Province, China.

The following reagents and materials were used in this study: the pMD-18 T vector (TaKaRa, China, Cat# 6011), the RNA simple Total RNA Extraction Kit (TIANGEN, China, Cat# DP419), the HiScript II Q RT SuperMix for qPCR (+gDNA wiper) reverse transcription kit (Vazyme, China, Cat# R223–01), the DNA Purification and Recovery Kit (TIANGEN, China, Cat# DP204–02), and DH5α competent cells (TOLOBIO, China, Cat# CC96102–02).

### 2.2 Samples

The affected flock consisted of 4,600 Tianfu Ma ducks aged 26 days. The ducks had been administered a sequential immunization protocol consisting of: a combined antibody against duck hepatitis virus type 1 and type 3 (procured from Pulike Biological Engineering Co., Ltd.) and goose parvovirus (gosling plague) on day 1, duck hepatitis virus type 1 and type 3 antibody (procured from Pulike Biological Engineering Co., Ltd.) on day 3, reovirus antibody on day 5, a triple vaccine against Newcastle disease virus, avian influenza virus, and fowl adenovirus (procured from Qingdao Yebio Bioengineering Co., Ltd.) on day 7, an H5 + H7 subtype avian influenza inactivated vaccine (also procured from Qingdao Yebio Bioengineering Co., Ltd.) on day 10, and a further dose of duck hepatitis virus type 1 and type 3 antibody (from Pulike Biological Engineering Co., Ltd.) on day 14. The disease manifested as an acute, fatal syndrome with a cumulative incidence (4.57%; *n* = 210) equal to the mortality rate. Affected ducks manifested opisthotonos, a characteristic neurological sign. Post-mortem examination revealed lesions characteristic of duck viral hepatitis, including hepatomegaly with severe hemorrhage and parenchymal friability, distended gallbladder, renomegaly exhibiting a mottled surface, and dark-colored intestinal contents.

### 2.3 Virus isolation and identification

Diseased duck liver tissues were divided into two samples, homogenized, and subjected to freeze–thaw cycles, followed by centrifugation to collect the supernatant. Nucleic acid was extracted from tissue samples and amplified by PCR with DHAV-3 specific primers (Table 1), after which positive samples were inoculated into 15-day-old non-SPF goose embryos. The positive supernatant was then filtered through a 0.22 μm membrane. The filtrate was inoculated into 15-day-old non-SPF goose embryos via the allantoic cavity (0.3 mL per embryo). A control group was inoculated with an equivalent volume of sterile normal saline.

**Table 1:** Primer sequences for identification and whole-genome amplification of DHAV-3.

Primer name	Primer (5′ → 3′)	Size (bp)	Notes
DHAV-3 F	TACGCCAGCCACTCATCT	400	Detection primer
DHAV-3 R	TGGTCAGGCACTGGCAAAT		
DHAV-3 1F	TTTGAAAGCGGCTGTGGTGTAG	987	Whole-genome sequencing primers
DHAV-3 1R	ACTGTATAAAAACATCCAGGAGTGGC		
DHAV-3 2F	GCCCAGGGTGATAACATCTCA	987	
DHAV-3 2R	TCCATTAGAGACAGGGTACCAT		
DHAV-3 3F	GGCTAACACAGTAACCCCT	1,183	
DHAV-3 3R	TTCAATTTCTAGATGGAGCTCAAAGGCAA		
DHAV-3 4F	ACTTTTGGCAGGTTGTATATTTGGA	1,166	
DHAV-3 4R	AAACCCAATGCTGCAGCCC		
DHAV-3 5F	GAATCACTTGTTCGTGCCTGT	1,264	
DHAV-3 5R	TCCAAATACCAAGAGGTTCGGGTC		
DHAV-3 6F	GTGCAAAAAATGTTCAATGGTGGT	990	
DHAV-3 6R	ACATTGTATCCAAATCACTTAGCCTA		
DHAV-3 7F	CCCCTTCAGAAATATAATCATAAAGGT	1,054	
DHAV-3 7R	TTAGGTTTCCTCCAACAAGGGCTA		
DHAV-3 8F	GCACGACATCATGCCAAACA	1,144	
DHAV-3 8R	GGTACAGACAAAACACAATCA		
DHAV-3 9F	GACGAGATATGGAAGGTGGAA	926	
DHAV-3 9R	TTTTTAGGGTGGGGAGGAATAG		

Embryos that died within 24 h post-inoculation were discarded. The remaining embryos were examined every 8 h for 7 days. Surviving embryos were euthanized by storing them at 4 °C. Liver, spleen, and allantoic fluid were collected from deceased embryos and stored at −80 °C for further analysis.

To confirm the successful isolation of DHAV-3, nucleic acid was extracted from the harvested allantoic fluid, liver, and spleen tissues of non-SPF goose embryos, followed by PCR amplification using DHAV-3 specific primers (Table 1).

### 2.4 Primer design for DHAV-3 detection and whole-genome amplification

DHAV-3 detection primer was designed using SnapGene and Primer Premier 5 software. For whole-genome amplification of DHAV-3, nine pairs of primers were designed using SnapGene and Primer Premier 5 software. Based on the reference strain sequence (GenBank accession no. MH752740), a segmented amplification strategy was followed. These primers were used to amplify the complete viral genome (Table 1). All the above primers were synthesized by Shangya Biotechnology Co., Ltd.

### 2.5 Molecular cloning and sequencing

The nine amplified fragments of DHAV-3 underwent PCR amplification and gel extraction. Subsequently, they were ligated into the pMD-18 T vector and transformed into DH5α competent cells. The transformed cells were spread onto LB solid medium supplemented with ampicillin for cultivation. Positive clones, identified through PCR screening, were sent to Shangya Biotechnology Co., Ltd. for sequencing.

### 2.6 Sequence assembly and phylogenetic analysis

Genome assembly was carried out using the SeqMan Pro (v7.1.0) module of the DNASTAR Lasergene 7.1 package. Reference sequences of DHAV strains, encompassing both domestic and international isolates (refer to Table S1 for accession numbers), were retrieved from the NCBI GenBank database. Sequence homology for the complete genome and the VP1 gene was analyzed using the MegAlign (v7.1.0, DNASTAR). Phylogenetic trees were constructed with MEGA 7 software.

Furthermore, the tertiary protein structures were predicted by AlphaFold3, and the locations of amino acid mutations were visualized and annotated using PyMOL (v3.0.3). Finally, sequence homology heatmaps were generated, and all figures were compiled and typeset using Origin 2024.

## 3 Results

### 3.1 Isolation and identification of virus

**Figure 1:**
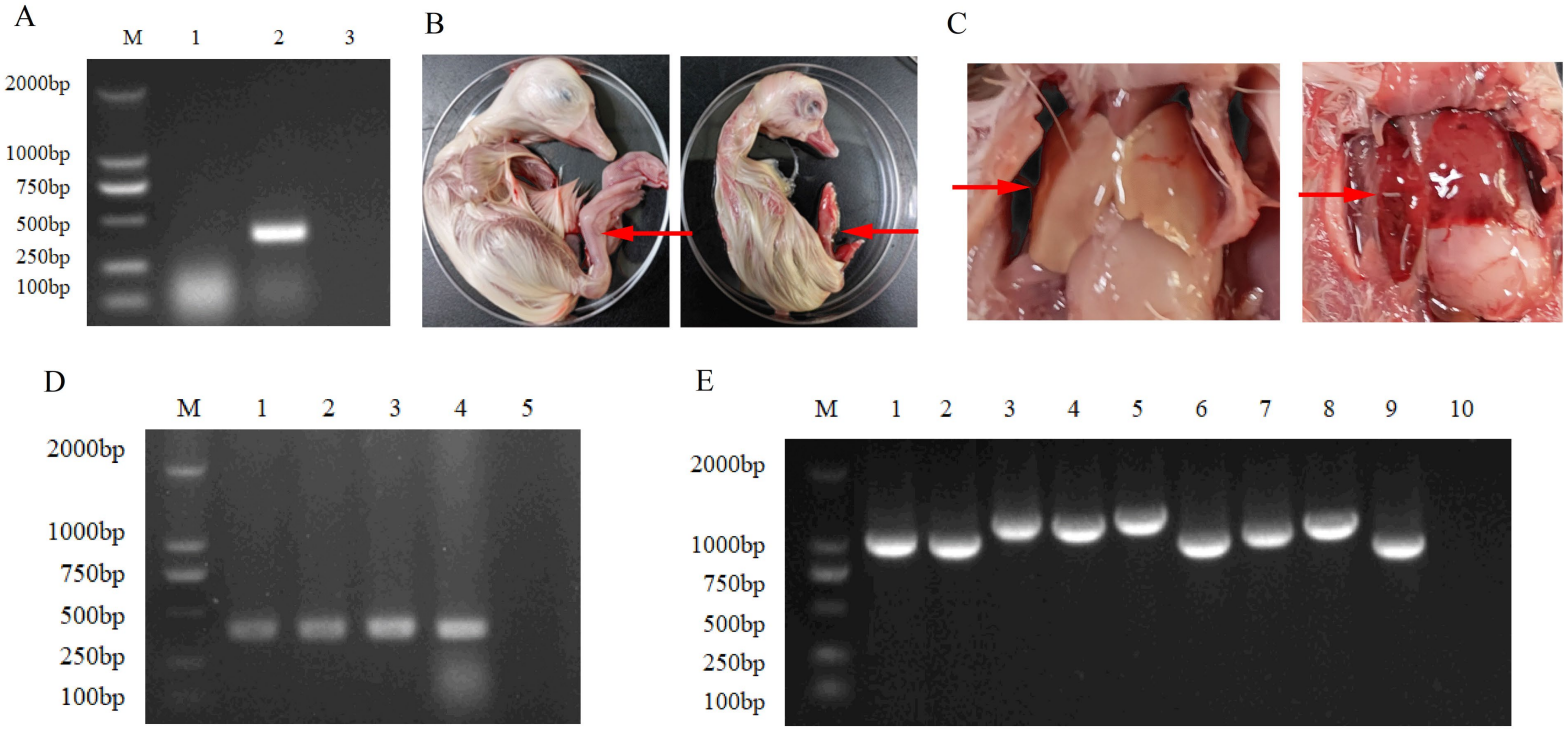
Isolation and identification of DHAV-3 strain. **(A)** PCR detection of DHAV-3 in clinical liver samples. Lane M: DL2000 DNA marker; Lane 1: Sample 1; Lane 2: Sample 2 (showing the expected amplification fragment); Lane 3: Negative control. **(B)** Gross morphology of goose embryos at 5 days post-inoculation. The left panel: Normal control embryo; the right panel: Inoculated embryo showing reduced size and diffuse congestion. **(C)** Pathological changes in embryonic livers. The left panel: Normal liver architecture in control embryo; the right panel: Affected embryo exhibiting hepatomegaly, congestion, and hemorrhagic foci. **(D)** PCR confirmation of DHAV-3 replication in embryonic tissues. Lane M: DL2000 DNA marker; Lanes 1–3: Allantoic fluids from inoculated embryos; Lane 4: Liver-spleen homogenate from deceased embryo; Lane 5: Negative control. **(E)** Amplification of the complete DHAV-3 genome using nine overlapping primer sets. Lane M: DL2000 DNA marker; Lanes 1–9: Amplification products of the nine genomic segments; Lane 10: Negative control.

Following the processing of the clinical samples, an RT-PCR product of the expected size for the target gene was successfully amplified from sample 2 (Figure 1A). Based on PCR screening (primers are listed in Supplementary Table S2), other infections—including duck plague, adenovirus, avian influenza, reovirus, and Newcastle disease—were ruled out.

For sample 2, after inoculation into three 15-day-old non-SPF goose embryos, one embryo died five days post-inoculation. The dead embryo was smaller in size and showed diffuse congestion (Figure 1B). Gross examination revealed extensive hepatic congestion with hemorrhagic foci along the liver margin (Figure 1C). Nucleic acid was extracted from the allantoic fluid, as well as from the harvested liver and spleen. Subsequent PCR amplification yielded a band consistent with the expected size of the target gene (Figure 1D). Consequently, a single strain of DHAV-3 was obtained.

### 3.2 Amplification of the genome

Nine specific primers were designed based on the complete genomic sequence of DHAV-3. Using these primers, PCR amplification yielded nine distinct target bands, each corresponding to the expected sizes (Figure 1E). Moreover, the band size of the recombinant plasmid observed after electrophoresis was consistent with that of the target fragment presented in Figure 1C, which confirmed the successful cloning.

### 3.3 Whole gene sequencing results

These sequence data have been submitted to the GenBank database under accession number PP977088 for the complete genome sequence of the isolated DHAV-3 strain HNAY2024.

Whole-genome sequencing determined the genome to be 7,779 bp, which comprises a 653 bp 5′ UTR, a single 6,755 bp open reading frame (ORF; nucleotides 653–7,408), and a 371 bp 3′ UTR. The overall nucleotide composition was 28.6% A, 29.7% T, 19.7% C, and 22.0% G.

The ORF encodes a polyprotein of 2,251 amino acids, with a predicted secondary structure composition of 36.78% *α*-helices, 13.64% extended strands, and 49.58% random coils.

#### 3.3.1 Whole-genome genetic similarity analysis of isolated strains

Nucleotide sequence similarity analysis conducted using MegAlign indicated that the isolated strain HNAY2024 shared 72.6 to 99.9% identity with the reference strains. Specifically, it had 72.6 to 72.9% similarity with DHAV-1 reference sequences, 77.8% with DHAV-2 reference sequence, and 91.9 to 99.9% with other DHAV-3 reference strains.

**Figure 2:**
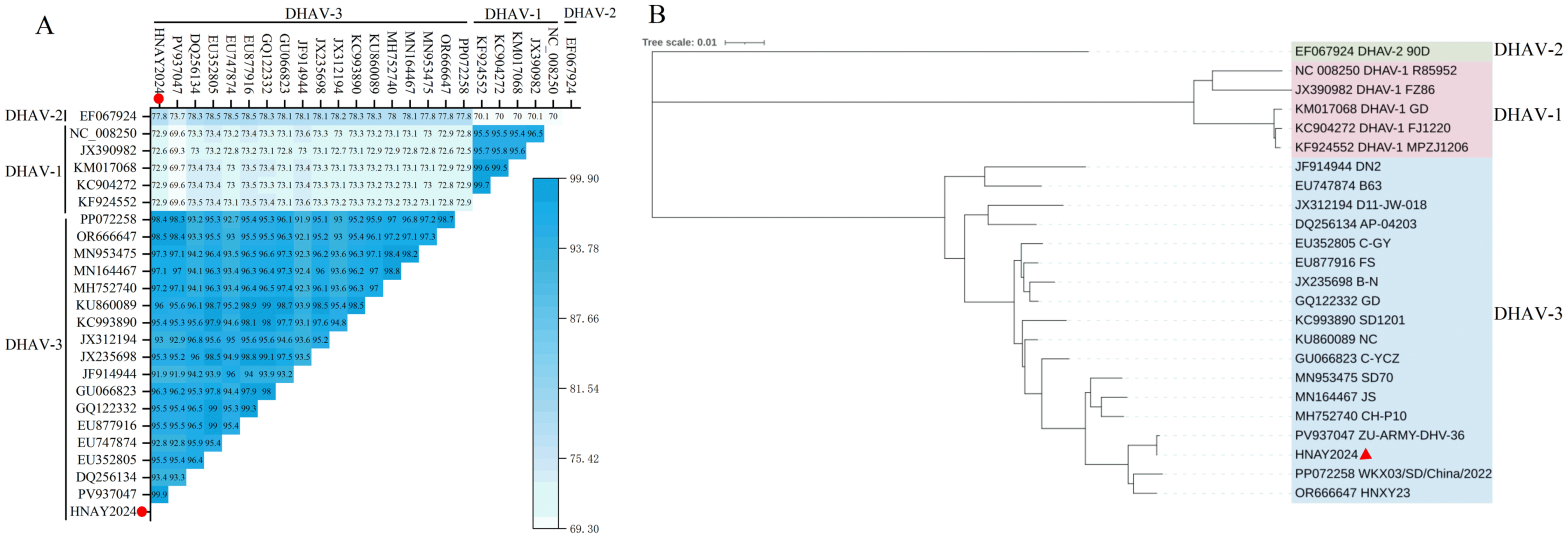
Genetic analysis of the isolated DHAV-3 strain. **(A)** Triangular heatmap depicting nucleotide identity across the complete genome between the isolated strain (HNAY2024, this study, marked with red circles) and reference strains. Values represent percentage identity calculated using MegAlign software. **(B)** Phylogenetic tree based on complete genome sequences constructed using the Neighbor-Joining method in MEGA 7. The isolated strain in this study (HNAY2024, PP977088) is marked with a red triangle.

Among the DHAV-3 reference strains, this isolate exhibited the highest nucleotide sequence identity (99.9%) with the 2025 Egyptian isolate ZU-ARMY-DHA-36. Among the Asian reference strains, the highest similarities were observed with strain HNXY23 (98.5%), which was isolated in Henan in 2023, and strain WKX03/SD/China/2022 (98.4%), which was isolated in Shandong in 2022. In contrast, the lowest similarity (91.9%) was found with strain DN2, which originated from Vietnam in 2011 (Figure 2A).

#### 3.3.2 Genetic relationship analysis of whole-genome of isolated strains

Phylogenetic analysis based on the complete genome sequence was performed using MEGA 7. The results demonstrated that the isolate obtained in this study belongs to the DHAV-3 genotype and clusters most closely with strains ZU-ARMY-DHA-36, HNXY23 and WKX03/SD/China/2022, forming a well-supported clade with these strains (Figure 2B).

#### 3.3.3 Similarity and genetic relationship analysis of VP1 protein in isolates

**Figure 3:**
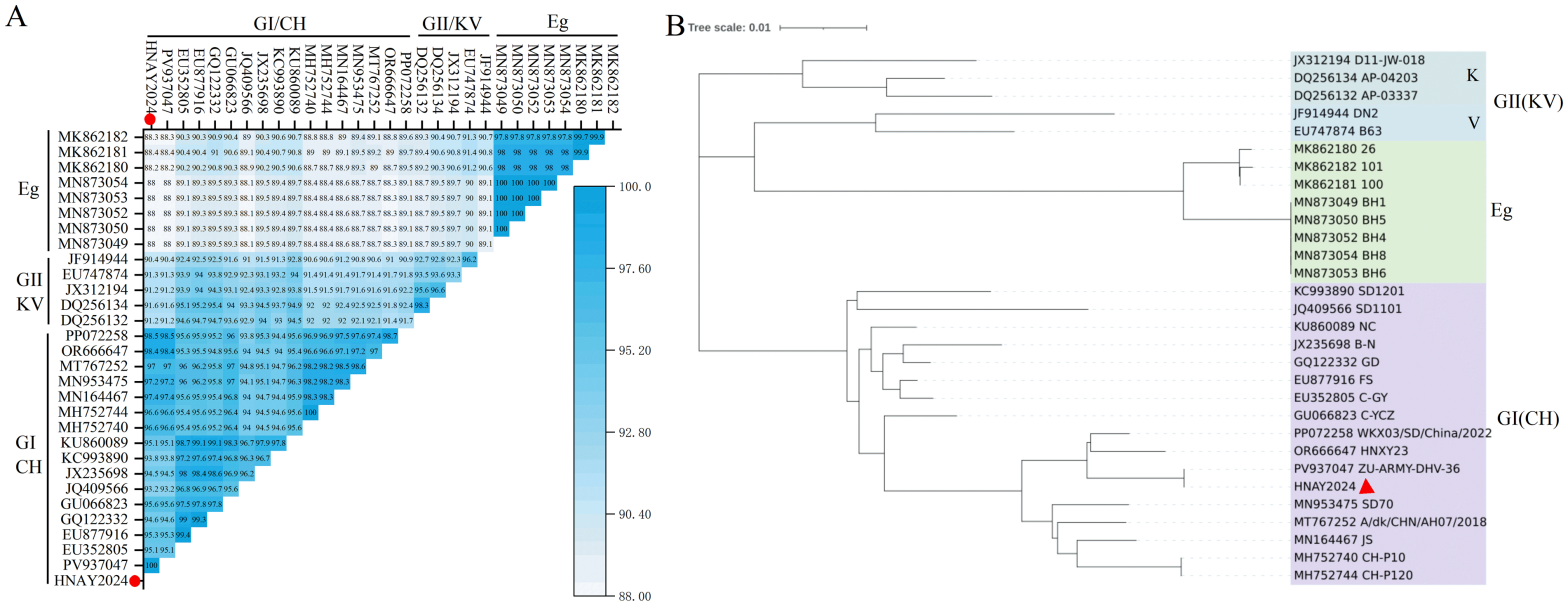
Genetic and structural characterization of the VP1 protein from the isolated DHAV-3 strain. **(A)** Triangular heatmap depicting nucleotide identity across the complete VP1 gene between the isolated strain (HNAY2024, this study, marked with red circles) and reference strains. **(B)** Phylogenetic tree constructed based on VP1 nucleotide sequences using the Neighbor-Joining method. The isolated strain in this study (HNAY2024, PP977088) is marked with a red triangle.

Comparative analysis indicated that the VP1 nucleotide sequence of the isolated strain exhibits 88.0 to 100% identity with reference DHAV-3 strains (Figure 3A). The highest identity (100%) was detected with strain ZU-ARMY-DHA-36, whereas the lowest identity (88.0%) was identified with the Egyptian isolates (BH1, BH4, BH5, BH6, BH8), which belong to the E group.

Based on phylogenetic analysis of the VP1 gene, the isolate was classified into genotype I (GI) of DHAV-3. Phylogenetic analysis further demonstrated that the VP1 sequence of the current isolate clusters within the same clade as strain ZU-ARMY-DHA-36 and displays a close genetic relationship with strains HNXY23 and WKX03/SD/China/2022 (Figure 3B).

#### 3.3.4 Whole-genome mutation site analysis

**Figure 4:**
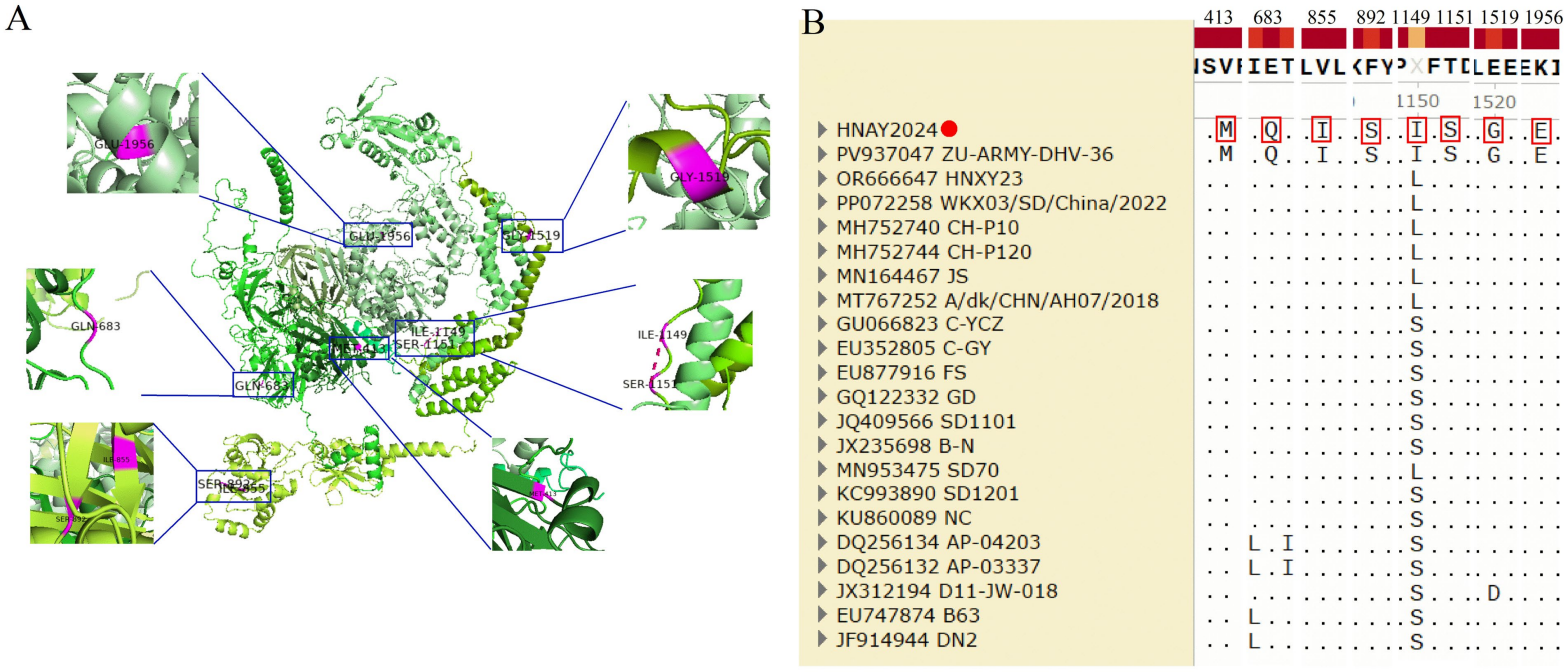
Tertiary structure and mutation sites of the HNAY2024 isolate. **(A)** Structural model of the viral polyprotein predicted by AlphaFold3. Mutated residues are highlighted as purple spheres. The viral proteins are distinguished by color: VP0 (green), VP3 (forest green), VP1 (teal), 2A (lemon), 2B (chartreuse), 2C (lime), 3A (split-pea green), 3B (lime green), 3C (smoky), 3D (pale green). **(B)** Mutations identified in HNAY2024 (marked by red circles) are compared against the reference sequence.

The HNAY2024 isolate harbors eight amino acid substitutions relative to the reference strains: V413M (VP3-157), E683Q (VP1-190), V855I (2A-122), F892S (2A-159), S1149I (2B-111), T1151S (2B-113), E1519G (3A-29), and K1956E (3D-158). However, these amino acid positions are identical to those of the Egyptian isolate ZU-ARMY-DHA-36 (Figures 4A,B).

## 4 Discussion

Duck hepatitis is one of the major infectious diseases that seriously threaten ducks worldwide. In China, although farms have implemented biosafety measures, including vaccination and the use of antibodies, duck hepatitis still occurs and spreads in local areas (especially in intensive breeding zones) (Zhou et al., 2022). This is due to factors such as China’s vast territory, large duck population, frequent transportation of ducks and their products, potential cross-transmission from wild waterfowl, and management loopholes on farms (Zhou et al., 2022; Zhao et al., 2024; Wen et al., 2018). It causes severe damage to waterfowl production. Moreover, since there are multiple serotypes of the virus with extremely low antigen cross-reactivity (Wu et al., 2020; Liang et al., 2020) and the virus’s VP1 protein has a high evolutionary rate, circulating epidemic strains often do not match vaccine strains. As a result, vaccination and egg yolk antibody therapy frequently fail to provide satisfactory protective or therapeutic effects (Truong and Cheng, 2022). To prevent and control this disease, organized and purposeful continuous field monitoring is particularly necessary. This study was conducted during the close monitoring of duck hepatitis detection in some parts of Henan Province in central China. The results showed that a potentially novel DHAV-3 strain with typical pathological features was successfully isolated and identified based on the mortality of inoculated embryos that occurred within 5 days post-inoculation (Shawki et al., 2024), as well as its genome analysis. The dead embryos showed clear pathological signs, including significant stunting and generalized redness (Figure 1B), and necropsy findings in the liver (Figure 1C).

Whole-genome phylogenetic analysis revealed that HNAY2024 belongs to the DHAV-3 genotype and clusters closely with the ZU-ARMY-DHA-36, HNXY23 and WKX03/SD/China/2022 strains. Geographically, the four strains were isolated from relatively distant locations. The HNAY2024 strain was isolated from northern Henan Province, China; ZU-ARMY-DHA-36 from Egypt; HNXY23 originated from Xinyang in southern Henan Province, China; and the WKX03/SD/China/2022 strain came from Shandong Province, China. These findings suggest that the evolution of DHAV-3 may not be strictly geographically restricted but instead shows a stronger correlation with temporal factors, supporting previous reports that the genetic divergence of DHAV-3 is closely associated with isolation time (Ma et al., 2015). This may also indicate that frequent trade of ducks and duck products, interaction between domestic and wild flocks have broken geographical barriers, leading to the prevalence of such strains in China. Previous studies have also reached a similar conclusion (Zhou et al., 2022).

The VP1 gene of this strain consists of 715 nucleotides, encoding 238 amino acids. Phylogenetic analysis based on VP1 classified HNAY2024 into DHAV-3 genotype I (GI). Interestingly, among the Asian reference strains, although the three strains exhibited a close evolutionary relationship in the whole-genome phylogenetic tree, their key antigenic protein VP1 exhibited notable variations, and the VP1 protein of the target strain underwent specific modifications. The concordance between the VP1 and whole-genome phylogenetic trees confirms that VP1 is not only a critical antigenic region but also a reliable marker for reflecting the temporal (Ma et al., 2015) evolutionary pattern of DHAV-3.

The ORF of HNAY2024 was found to encode a polyprotein of 2,251 amino acids, consistent with the lengths documented for other DHAV-3 strains (Lan et al., 2025). Furthermore, it had eight amino acid substitutions compared to the Asian reference strains (Supplementary Table S1, Figure 4): V413M (VP3-157), E683Q (VP1-190), V855I (2A-122), F892S (2A-159), S1149I (2B-111), T1151S (2B-113), E1519G (3A-29), and K1956E (3D-158). The mutations are distributed across both structural and non-structural proteins, suggesting possible adaptations in antigenicity, replication fitness, and host interaction. The biological significance of these mutations requires further investigation. The identity of these amino acid positions with those of the Egyptian isolate ZU-ARMY-DHA-36 implies a possible introduction of the strain into Egypt. However, whether it was introduced into Egypt through migratory birds or international trade requires further epidemiological investigation.

The E683Q substitution at position 190 of the major capsid protein VP1 is of particular interest. The VP1 protein, a major antigenic determinant of DHAV-3, plays a critical role in viral pathogenicity, evolution, and virulence (Truong and Cheng, 2022). These mutation sites are primarily located in the C-terminal region of VP1, which represents a hypervariable region (HVR) that harbors major conformational epitopes. This region serves as the primary binding site for neutralizing antibodies, which is crucial for their virus-neutralizing function (Wen et al., 2018; Ma et al., 2015; Zhao et al., 2025; Kim et al., 2024; Liu and Kong, 2019; Zhang et al., 2018). Alterations within this region are likely to modulate viral antigenicity and host cell tropism. In this study, the virus isolate exhibited mutations within this critical VP1 region. Clinical data from the author show that although farm ducks were vaccinated with duck hepatitis vaccine or administered duck hepatitis virus type 1 and type 3 antibodies, they still contracted the virus at approximately 20–26 days of age, accompanied by severe mortality in the flock. This clearly differs from the classic clinical manifestations of duck hepatitis (which typically occur within 1 week of age), as supported by literature (Kim et al., 2024; Swayne et al., 2020). Moreover, similar clinical cases have been continuously observed in duck flocks across multiple local farms. Interestingly, prior to 2019, outbreaks of duck viral hepatitis predominantly occurred in ducks within 2 weeks of age. However, after 2019, a marked delay in the age of onset was observed, with the majority of cases occurring in ducks older than 2 weeks, and some even extending to 5–6 weeks of age. Accumulating evidence suggests that this shift in onset age may be attributed to two primary factors: the extensive application of duck hepatitis virus antibodies, and the increased pathogenicity resulting from viral variation. The administration of antibodies appears to provide partial protection in the early stage, thereby delaying the onset of disease. Nevertheless, infection may still occur once the specific antibody titers in ducks decline and eventually wane. The increasing incidence of disease in older ducks implies that the virulence of circulating viral strains may have intensified. In conjunction with genomic analyses, it cannot be ruled out that such enhanced virulence is closely associated with viral evolution, although further studies are warranted to validate this hypothesis. These findings support the idea that the VP1 mutation may lead to immune evasion or immune failure, resulting in hepatitis outbreaks in production. This also suggests that HNAY2024 is a newly emerging, locally predominant, and potentially novel field variant. Although this variation may reflect an immune evasion mechanism or an adaptive alteration to enhance receptor utilization, this hypothesis necessitates further validation through serological neutralization assays (Song et al., 2019; Truong and Cheng, 2022).

The V413M mutation in the structural protein VP3 represents a strong candidate site for adaptive evolution, potentially enhancing viral fitness by improving capsid stability or fine-tuning host cell interactions (Zhang et al., 2025). Among the non-structural proteins, the clustered S1149I and T1151S mutations in the 2B protein suggest potential functional adaptation. As a viroporin, the 2B protein disrupts cellular calcium homeostasis and induces incomplete autophagy; these adjacent substitutions could conceivably enhance its capacity to modulate the host cellular environment, thereby facilitating viral replication (Liu et al., 2021). Similarly, the E1519G mutation in the 3A protein, which participates in viral replication complex formation, may alter its interactions with host factors or membranes (Li et al., 2024). Furthermore, the K1956E substitution at position 158 of the RNA-dependent RNA polymerase (3D) warrants particular attention. As the catalytic core of the viral replication machinery, even this conservative substitution of a charged residue could subtly modulate polymerase fidelity, processivity, or interactions with the VPg primer, ultimately influencing viral replication kinetics and genetic diversity (Li et al., 2024).

Although this isolate was obtained from domestic ducks, the identified mutational profile, particularly in key proteins like VP1 and 3D, raises important questions regarding its potential ecological impact. The observed genetic changes, which may enhance viral adaptability and replication efficiency in domestic hosts (Kim et al., 2024), could theoretically alter the virus’s host range or transmissibility. Given that wild ducks (e.g., mallards, *Anas platyrhynchos*) often share aquatic habitats with farmed ducks, they could be exposed to spillover events. If adaptive mutations like those in VP1 (affecting antigenicity and receptor usage) or 3D (affecting replication fidelity) confer an advantage in a new host environment, wild duck populations could potentially serve as asymptomatic reservoirs or experience altered pathogenicity. This underscores the necessity for future surveillance studies to directly investigate the presence, prevalence, and genetic characteristics of DHAV-3 in sympatric wild duck populations. Understanding the interplay between viral evolution in domestic settings and potential spillover into wildlife is crucial for comprehensive disease ecology and biosecurity.

In conclusion, the mutation profile of HNAY2024, particularly within the immunodominant VP1 protein and key replication-associated proteins such as 2B and 3D, suggests a viral lineage that has potentially evolved under selective pressure to acquire altered antigenic characteristics and enhanced replication efficiency.

## Data Availability

The datasets presented in this study can be found in online repositories. The names of the repository/repositories and accession number(s) can be found in the article/Supplementary material.
