# Supplementary material for: Isolation, characterization and whole-genome analysis of a potentially novel strain of duck hepatitis A virus type 3 from a vaccinated duck flock in China
Source: Front Microbiol. 2026 Mar 25;17:1775404. doi: 10.3389/fmicb.2026.1775404 (PMC13057283; doi:10.3389/fmicb.2026.1775404)
Supplement: Supplementary file 1 [file Data_Sheet_1.pdf]

Table S1 Reference genome information

Type	Strain	Login ID	Source	Time	
	MH752740	CH-P10	Sichuan, China	2018	
	DQ256132	AP-03337	South Korea	2005	
	GU066823	C-YCZ	Beijing, China	2009	
	EU747874	B63	Beijing, China	2008	
	JX312194	D11-JW-018	South Korea	2012	
	JX235698	B-N	Guangdong, China	2012	
	GQ122332	GD	Beijing, China	2009	
	MN953475	SD70	Jiangsu, China	2020	
	MN164467	JS	Heilongjiang, China	2019	
	OR666647	HNXY23	Henan, China	2023	
	MH752744	CH-P120	Sichuan, China	2018	
DHAV-3	KC993890	SD1201	Shandong, China	2013	
	DQ256134	AP-04203	South Korea	2005	
	EU352805	C-GY	Beijing, China	2010	
	JF914944	DN2	Vietnam	2011	
	EU877916	FS	Guangdong, China	2008	
	JQ409566	SD1101	Shandong, China	2012	
	KU860089	NC	Vietnam	2016	
	PP072258	WKX03/SD/China/2022	Shandong, China	2024	
	MT767252	A/dk/CHN/AH07/2018	Shandong, China	2020	
	PV937047	ZU-ARMY-DHV-36	Egypt	2025	
	MN873049	BH1	Egypt	2020	
	MN873050	BH5	Egypt	2020	VP1
	MN873052	BH4	Egypt	2020	

	MN873053	BH6	Egypt	2020
	MN873054	BH8	Egypt	2020
	MK862180	26	Egypt	2020
	MK862181	100	Egypt	2020
	MK862182	101	Egypt	2020
	KF924552	MPZJ1206	Fujian, China	2014
	KC904272	FJ1220	Fujian, China	2013
DHAV-1	KM017068	GD	Guangdong, China	2014
	JX390982	FZ86	Fujian, China	2012
	NC_008250	R85952	the United States	2018
DHAV-2	EF067924	90D	Taiwan, China	2007

Table S2 Primer sequences for the differential exclusion of other pathogenic infections

virus name	Primer (5'→3')	Size(bp)
duck plague	GAA GGC GGG TAT GAT ATG TA<br>CAA GGC TCT ATT CGG TAA TG	446
adenovirus	CAA RTT CAG RCA GAC GGT<br>TAG TGA TGM CGS GAC ATC AT	897
avian influenza	CTT CTA ACC GAG GTC GAA ACG<br>AGG GCA TTT TGG ACA AAK CGT	244
reovirus	CCC ATG GCA ACG ATT TC<br>TTC GGC CAC GTC TCA AC	399
Newcastle disease	GGA GGA TGT TGG CAG CAT T<br>GTC AAC ATA TAC ACC TCA TC	310
